# Inter-individual predictors of pain inhibition during performance of a competing cognitive task

**DOI:** 10.1038/s41598-020-78653-z

**Published:** 2020-12-11

**Authors:** V. Tabry, T. A. Vogel, M. Lussier, P. Brouillard, J. Buhle, P. Rainville, L. Bherer, M. Roy

**Affiliations:** 1 Faculty of Medicine, McGill University, Montreal, QC Canada; 2 Department of Psychology, McGill University, Montreal, QC Canada; 3 Centre de Recherche de l’Institut Universitaire de Gériatrie de Montréal (CRIUGM), Montreal, QC Canada; 4 Faculté de Médecine, Université de Montréal, Montreal, QC Canada; 5 Mila, Université de Montréal, Montreal, QC Canada; 6 University of Southern California, Los Angeles, USA; 7 Département de Stomatologie, Faculté de Médecine Dentaire, Université de Montréal, Montreal, QC Canada; 8 Montreal Heart Institute, Montreal, QC Canada; 9 Département de Medicine, Université de Montréal, Montreal, QC Canada; 10 Alan Edwards Centre for Research on Pain (AECRP), McGill University, Montreal, QC Canada

**Keywords:** Pain, Psychology, Risk factors

## Abstract

The main function of pain is to automatically draw attention towards sources of potential injury. However, pain sometimes needs to be inhibited in order to address or pursue more relevant tasks. Elucidating the factors that influence how people manage this relationship between pain and task performance is essential to understanding the disruptive nature of pain and its variability between individuals. Here, 41 healthy adults completed a challenging working memory task (2-back task) while receiving painful thermal stimulations. Examining the trial-by-trial relationship between pain perception and task performance revealed that pain’s disruptive effects on performance were mediated by self-reported pain intensity, and that the analgesic effects of a competing task were influenced by task performance. We found that higher pain catastrophizing, higher trait anxiety, and lower trait mindfulness were associated with larger trade-offs between pain perception and task performance, suggesting that these psychological factors can predict increased fluctuations between disruption by pain and analgesia from a competing task. Altogether these findings provide an important and novel perspective on our understanding of individual differences in the interplay between pain and ongoing task performance.

## Introduction

Pain acts as an alarm system; it rapidly disrupts ongoing activities to draw our attention towards sources of potential injury^1^. However, attending to pain may not always be the top priority, and thus our perception of pain may be inhibited to allow attention to be directed to more important tasks^2^. Together these observations suggest that pain’s disruptive effects on attention and the analgesic effects of a competing task are inversely correlated processes^3^. This negative relationship between pain perception and task performance can be explained by the limited resource model of attention^4^, which posits that different environmental stimuli compete for access to limited attentional resources, potentially leading to a trade-off or compromise between the two processes. However, these outcomes have rarely been studied in combination (see Supplementary Table S1 for review). In fact, difficulties downregulating pain in favour of a more valued goal may constitute a perpetuating factor for further pain, thereby increasing the likelihood of pain chronicity and associated cognitive deficits^5,6^.

An important question that remains to be investigated is which psychological traits affect this proposed trade-off, and whether this renders some individuals more vulnerable to interference by pain, and others more likely to experience reduced pain when performing a competing task. Identifying these individual factors can be challenging, however, as true differences between individuals can be obscured by differences in baseline pain sensitivity and cognitive ability. A key feature of our experimental design was therefore to account for the confounding factors of pain sensitivity and cognitive ability by individually calibrating noxious stimulus intensities and task difficulty to yield matched baseline levels of pain perception and task performance across participants. Consequently, any remaining between-person differences in pain interference and task-induced analgesia could be interpreted as reflecting the true variation in one’s propensity to favour either pain or performance when the two are in competition with one another.

The existing literature is additionally limited in detecting these differences, as most studies average subjective pain ratings and task performance across a number of trials over several minutes, thereby neglecting the highly dynamic nature of attentional processes^3,7^. For example, trial-by-trial (i.e., between-trial) fluctuations in attention may result in task-related analgesia for some trials and pain-related interference of performance for others, depending on one’s ability and disposition to prioritize the task efficiently and continuously. Similar to shifts on a balance scale, a model of limited resources^4^ posits that trials where more resources are allocated towards the task, performance is higher but pain processing is lower. Conversely, when more resources are allocated towards pain processing, higher pain is felt but performance is lower^3^. Whereas it may seem that maximizing allocation of attentional resources towards task performance is optimal in laboratory settings, pain is evolutionary designed to prevent injury in real life settings and can interrupt this process^1^. The dynamic competition between pain perception and concurrent goals therefore leads to a trade-off between the two processes. However, the role of pain-related psychological traits in biasing the balance of resources towards one or the other remains to be investigated.

Previous experimental studies have found that psychological traits predicting sensitivity to threatening stimuli, such as pain catastrophizing and fear of pain, amplify the overall disruptive effects of pain on cognition^8–11^. However, the effects of these traits on task-related analgesia have been studied less frequently, and available studies have shown mixed results: one study reported greater task analgesia in pain catastrophizers relative to controls^12^, while another study found less analgesia in pain catastrophizers^13^. The present study aimed to elucidate the role of pain catastrophizing in the competition between pain processing and concurrent task performance for access to limited cognitive resources. Moreover, based on previous findings^14,15^, we also measured how trait anxiety and trait mindfulness^16^ influenced these effects. Trait anxiety appears to affect one’s attentional focus and evaluation of pain in the context of distraction^17,18^, while mindfulness has been shown to mitigate the effects of pain catastrophizing on pain-related interference^19^ and conditioned fear learning^14^. We therefore posited that it would be negatively related to the effects of anxiety and pain catastrophizing in our pain–task paradigm.

## Results

**Figure 1:**
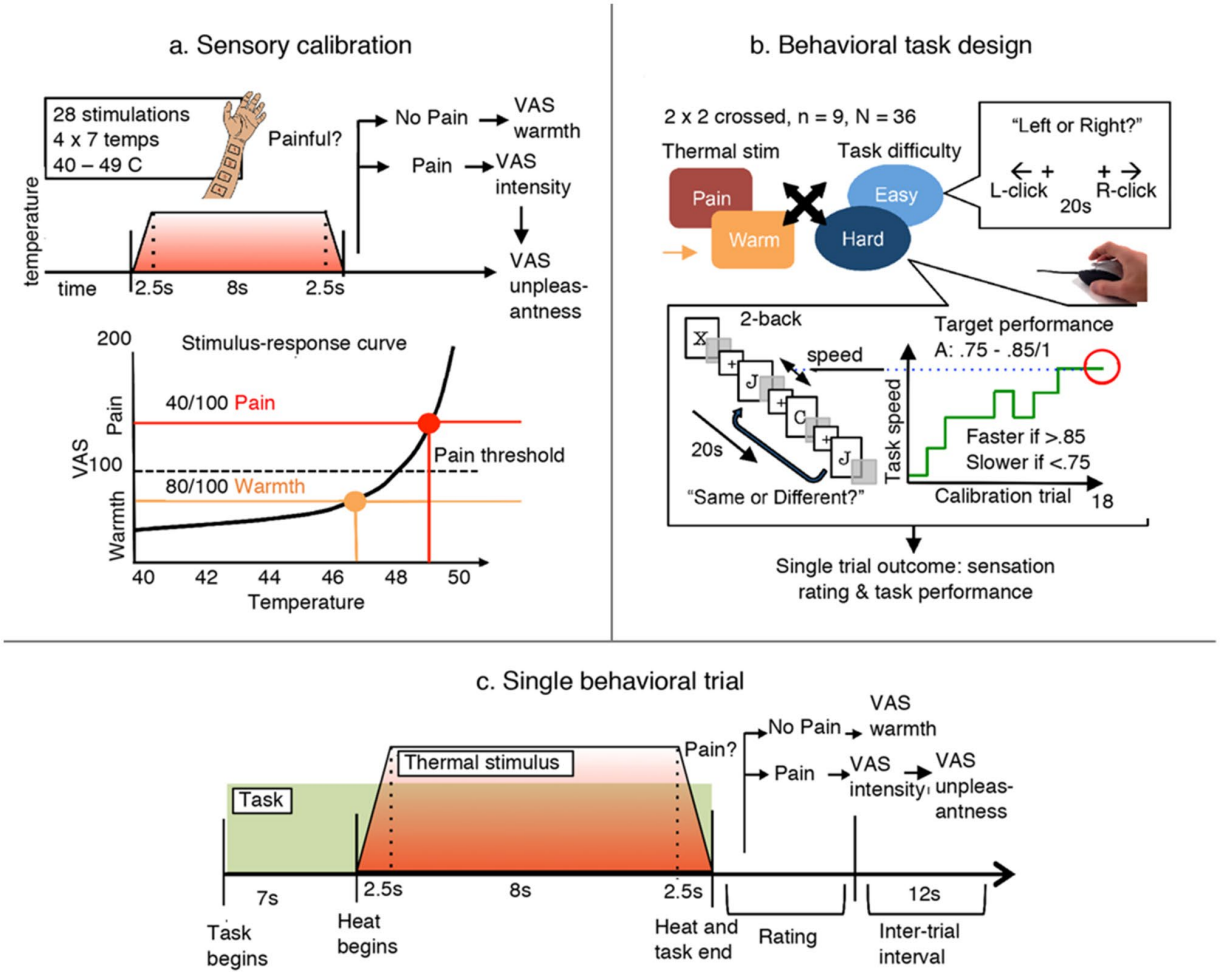
Sensory calibration and behavioral procedure. (**a**) Application of thermal stimuli and evaluation of warmth and pain on rating scales. Ratings were fitted to an exponential stimulus–response curve and individual warm and pain stimulation temperatures were derived. (**b**) Within-subjects complete crossing of two task difficulties (easy; difficult) and two thermal stimuli (Warm; Pain). The difficulty of the hard task (2-back) was individually calibrated over 18 trials using a staircase method, stabilizing performance between A = 0.75 and A = 0.85. (**c**) Timeline of a single behavioral trial. VAS: visual analogue scale.

Figure 1 depicts a summary of the methods. After data collection was complete, data from four participants were excluded from analysis due to the following: one for missing data (> 50% of trials) due to technical problems, two for inability to tolerate more than 50% of painful behavioral trials, and one for chance-level 2-back performance during the second testing session of the experiment. Data from the remaining 41 participants (21 females; mean age 24.2 years, range 19–36, SD = 4.5; mean education 17.2 years, range 12–27, SD = 3.0) were thus retained for our analyses.

### Descriptive statistics for sensory calibration, psychological factors, and task difficulty calibration

Descriptive statistics for the thermal sensory calibration, psychological factors, and 2-back task calibration are presented in Table 1. Warm temperature, Pain temperature, and pain threshold were highly correlated with one another, as expected (*p*s < 0.001). Pain catastrophizing was positively correlated with trait anxiety, while both were negatively correlated with mindfulness, also in line with our expectations (*p*s < 0.01). There was no significant correlation between the parameters for the 2-back task and sensory calibration procedures and our psychological variables of interest, indicating that any outcomes predicted by these variables would be unrelated to baseline differences in stimulation temperature or presentation speed in the cognitive task. As expected, performance was significantly higher in the easier LR task (Median > 0.99) compared to the more demanding 2-back task (Median = 0.85; *z* = 5.58, *p* < 0.001, *r* = 0.62). Pain (i.e., high heat) stimuli were perceived as significantly more painful than Warm (i.e., low heat) stimuli during both tasks (2-back: *t*(40) = 19.47, *p* < 0.001, Cohen's *d* = 3.04; LR: *t*(40) = 23.81, *p* < 0.001, Cohen's *d* = 3.72).

**Table 1:** Descriptives and intercorrelations of calibration parameters and psychological and cognitive factors.

Measure	Mean (SD)	1	2	3	4	5	6	7
**Sensory calibration**								
1. Pain threshold (°C)	46.20 (1.24)	–	0.99***	0.91***	0.16	0.06	0.14	− 0.10
2. Warm temp. (°C)	45.04 (1.52)		–	0.84***	0.18	0.10	0.08	− 0.07
3. Pain temp. (°C)	47.91 (0.82)			–	0.07	0.01	0.26	− 0.15
**Psychological factors**								
4. PCS	14.02 (10.69)				–	0.43**	− 0.59***	0.05
5. STAI-T	39.34 (10.13)					–	− 0.66***	0.22
6. 4-FFMQ	103.22 (17.20)						–	− 0.20
**2-back calibration**								
7. task character interval (ms)	579 (346)							–

Means and Pearson product-moment intercorrelations are presented for behavioral task parameters, thermal stimulus calibration parameters, and psychological and executive functions measures. PCS = Pain Catastrophizing Scale, PCS sum score ranges from 0 to 52; STAI-T = State-trait Anxiety Inventory—Trait, STAI-T score ranges from 20 to 80; 4-FFMQ = Five-Facet Mindfulness Questionnaire (Observe dimension omitted from total), FFMQ Act with Awareness, Describe, Nonjudge, Nonreact sum score ranges from 32 to 160: ***p < 0.001, **p < 0.01.

### Group-level effects

**Figure 2:**
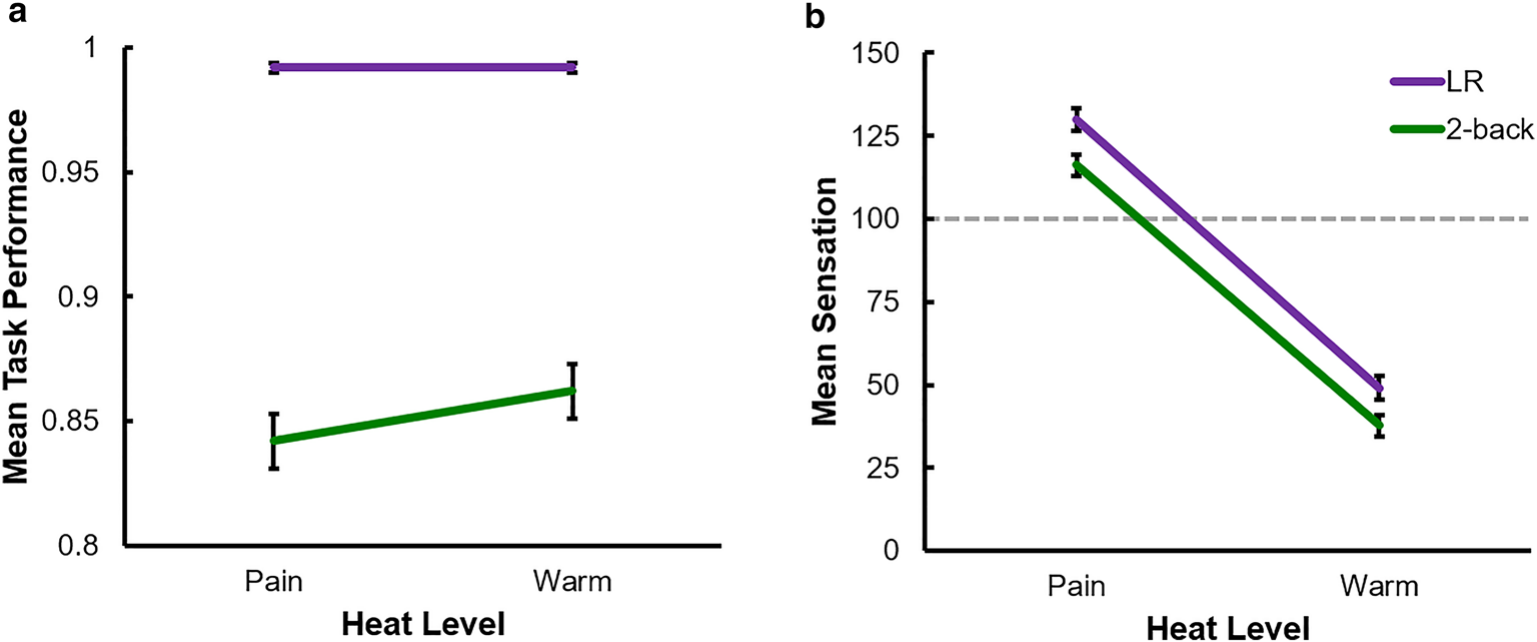
(**a**) Effects of task difficulty on mean performance and (**b**) effects of heat level on reported thermal sensation for the Left–Right and 2-back tasks. The grey line in (**b**) indicates pain threshold. Error bars represent standard error of the mean.

We first examined the effects of heat level on 2-back performance and found a near-significant difference in performance between the Warm (*M* = 0.86, *SD* = 0.07) and Pain (*M* = 0.84, *SD* = 0.07) conditions, *t*(40) = 1.76, *p* = 0.086, Cohen’s *d* = 0.28 (see Fig. 2a). There was no significant difference of performance on the LR task between the Warm (*M* = 0.99, *SD* = 0.02) and Pain (*M* = 0.99, *SD* = 0.01) conditions, *t*(40) = 0.04, *p* = 0.97, Cohen’s *d* < 0.01. Examining differences in ratings of thermal sensation (Fig. 2b), we found significantly lower reported sensation for the 2-back task (*M* = 116.17, *SD* = 25.95) compared to the LR task (*M* = 129.85, *SD* = 19.65) when participants received the painful temperature, *t*(40) = 4.41, *p* < 0.001, Cohen’s *d* = 0.59. Similarly, when receiving the non-painful, warm temperature participants reported lower sensation for the 2-back task (*M* = 37.74, *SD* = 21.99) compared to the LR task (*M* = 49.09, *SD* = 20.38), *t*(40) = 5.19, *p* < 0.001, Cohen’s *d* = 0.54. Mean reported sensation and task performance for all four conditions are displayed in Fig. 2. Furthermore, we examined the relationship between analgesia by the 2-back task, and task interference due to painful heat by examining the correlation between mean task-induced analgesia (MTA, mean sensation for Pain x LR task – mean sensation for Pain × 2-back) and mean pain-induced interference (MPI, mean performance for Warm × 2-back – mean performance for Pain × 2-back). There was a significant negative relationship between the two wherein those who displayed a higher analgesic effect of the difficult task exhibited lower interference by the pain stimulus (*r* =  − 0.35, *p* = 0.025). In addition, higher trait anxiety predicted lower MTA (*r* =  − 0.36, *p* = 0.019), but neither MTA nor MPI correlated with any of the other measured psychological traits (trait anxiety × MPI, *r* =  − 0.08, *p* = 0.64; pain catastrophizing × MTA, *r* =  − 0.20, *p* = 0.22; pain catastrophizing × MPI, *r* =  − 0.04, *p* = 0.79; mindfulness × MTA, *r* =  − 0.01, *p* = 0.94; mindfulness × MPI, *r* = 0.18, *p* = 0.27).

### Multilevel mediation models

**Figure 3:**
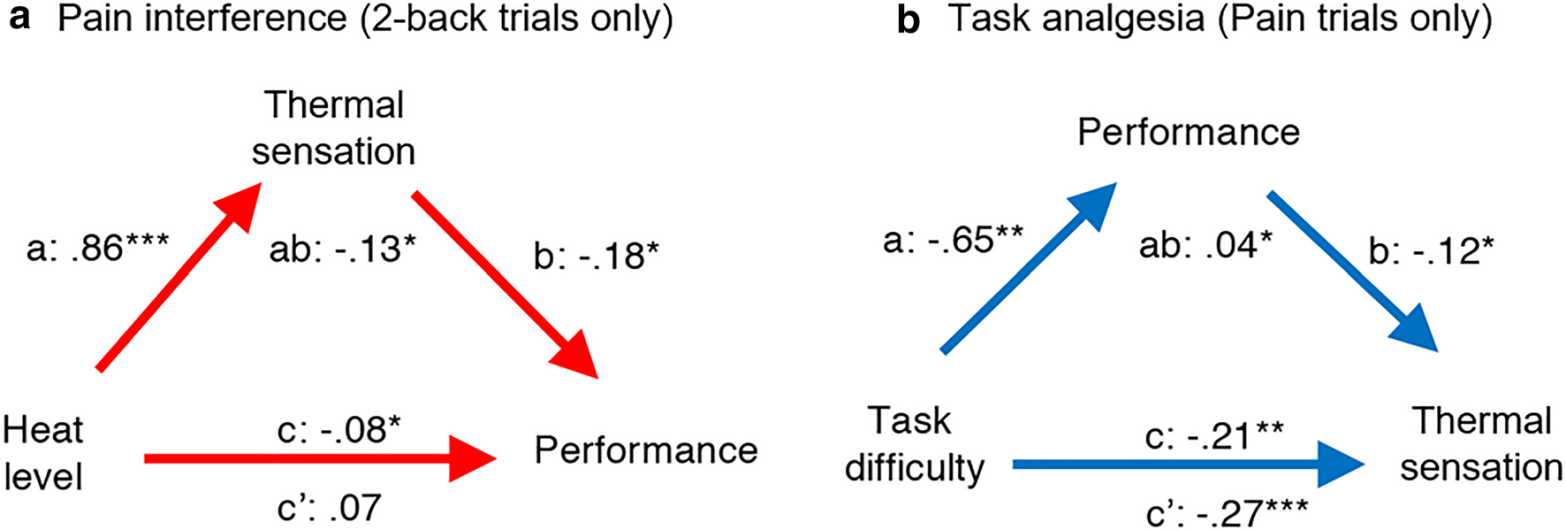
First-level mediation models of the effects of task difficulty on sensation and of heat level on task performance. In red (**a**), mediation of the effect of heat level on task performance by thermal sensation for 2-back trials only. In blue (**b**), mediation of the effect of task difficulty on reported sensation by task performance for Pain trials only. a, b, c, and c’ are mean standardized regression coefficients for the illustrated relationships; ***p < 0.001, **p < 0.01, *p < 0.05.

As the relationship between pain processing and ongoing task performance is highly dynamic, we used multilevel analyses to examine trial-by-trial fluctuations in thermal sensation and task performance. In this, variables at the level of a trial, such as task difficulty and heat level, are examined while accounting for differences between participants at the subject-level. Two distinct multilevel mediation models (see Fig. 3) were used to assess the trial-by-trial relationship between pain perception and task performance (see “Methods”). The first model examined whether the effects of heat level on task performance were mediated by trial-by-trial changes in thermal sensation, examining only 2-back trials, as performance was expectedly near ceiling for the easy LR task (pain interference model: heat level → thermal sensation → performance). The second model tested whether the effects of task difficulty on reported thermal sensation were mediated by trial-by-trial fluctuations in task performance, using only painful heat trials to examine changes in pain ratings specifically (task analgesia model: 2-back vs LR task → performance → thermal sensation). Therefore, these two different, albeit theoretically complementary, models aimed to examine our hypothesis of a bidirectional relationship between pain and performance from both directions. For each model, a subset of the data was used to better examine our hypotheses of interest; specifically, does thermal sensation mediate the effects of heat level on performance of a difficult cognitive task (2-back trials) and does task performance mediate the relationship between task difficulty and pain perception (painful heat trials only).

In the pain interference model (Fig. 3a), we first observed that heat level had a significant negative effect on task performance (total effect *c* path, *c* =  − 0.08, 95% CI [− 0.16, − 0.02], *p* = 0.049), suggesting that overall painful heat decreased performance of the 2-back task compared to non-painful warmth. Testing for mediation of this relationship, we found a significant positive effect of heat level on thermal sensation (*a* path, *a* = 0.86, 95% CI [0.82, 0.89], *p* < 0.001), indicating that more intense heat increased reported sensation, and a significant negative effect of sensation and task performance (*b* path, *b* =  − 0.18, 95% CI [− 0.33, − 0.01], *p* = 0.021), showing that greater perceived pain or warmth was associated with lower performance, after controlling for the heat level of the thermal stimulus. Together, the effect of heat level on performance mediated by sensation was significant (indirect effect, *ab* =  − 0.13, 95% CI [− 0.22, − 0.01], *p* = 0.031), suggesting that the relationship between heat level and task performance was explained through changes in reported thermal sensation. Specifically, at high (i.e., painful) levels of heat, subjective ratings of sensation increased which led to a decrease in task performance. Finally, the effect of heat level on task performance was no longer significant after controlling for thermal sensation (direct effect *c*′ path, *c′* = 0.07, 95% CI [− 0.08, 0.21], *p* = 0.33).

In the task analgesia model (see Fig. 3b), we found that the more difficult 2-back task significantly diminished reported sensation compared to the easier LR task, consistent with distraction analgesia (total effect *c* path, *c* =  − 0.21, 95% CI [− 0.31, − 0.13], *p* = 0.002). Examining the mediating relationship, we found that performance was lower for the 2-back task compared to the LR task (*a* path, *a* =  − 0.65, 95% CI [− 0.68, − 0.61], *p* = 0.006) and that pain ratings decreased with higher task performance (b path, *b* =  − 0.12, 95% CI [− 0.22, − 0.01], *p* = 0.035), after controlling for task difficulty. Together, the mediation of task difficulty on sensation through task performance was significant (indirect effect, *ab* = 0.04, *p* = 0.040, 95% CI [0.01, 0.11]) and in the opposite direction of the total effect (*c* path), indicating a suppressive effect of performance on thermal sensation. In other words, higher performance predicted decreased pain ratings, but this effect was partially counteracted by the increased task difficulty of the 2-back which reduces performance. Interestingly, after controlling for this mediating effect of performance, the analgesic effect of task difficulty (2-back vs LR) on sensation was stronger compared to the total effect (direct effect *c′*, *c′* =  − 0.27, *p* < 0.001, 95% CI [− 0.38, − 0.16]). The suppression effect of performance suggests that distraction analgesia from the 2-back task reduced pain through two mechanisms: a general analgesic mechanism consistent with previous reports of task-induced analgesia (see Supplementary Table S1) by which pain is reduced by engaging in a distractive, difficult task regardless of one’s performance, and another where the analgesia is dependent on maintaining good performance despite increased task difficulty. More simply, this may mean that the total analgesic effects of the 2-back task are strongest when participants push themselves to meet the demands of the challenging task and maintain high performance on the 2-back despite the increased difficulty.

Together, these findings provide evidence of a bidirectional relationship between task performance and pain perception. By examining the trial-by-trial relationship between these two processes, we observed that on trials where performance was high, pain ratings decreased (*b* path in task analgesia model). Conversely, on trials where more pain was felt (i.e., increased thermal sensation), performance appeared to decrease, suggesting that the attentional resources were allocated strategically between the two processes when they were in conflict. This bidirectional relationship supported our original hypothesis of a trade-off between pain and performance. However, we also aimed to understand how trait-level factors influenced this relationship.

### Moderation of trial-level mediation effects

The above findings suggest that the relationship between heat level and task performance can be explained through changes in thermal sensation, and that the relationship between task difficulty and thermal sensation can be suppressed by changes in task performance. Extending these models, we tested our main hypotheses of pain catastrophizing, trait anxiety, and trait mindfulness influencing the bidirectional relationship between pain and performance by separately including these variables as second-level moderators on each path in the two models (see Table 2 for model estimates; see Supplementary Fig. S1 for example diagram of model paths). In this way we were able to assess the change in path estimates based on one’s level of the given psychological variable (e.g., pain catastrophizing). In the pain interference model, the relationship of sensation predicting performance, after controlling for heat level (*b* path), was significantly positively moderated by trait anxiety and pain catastrophizing, and significantly negatively moderated by mindfulness. That is, higher pain catastrophizing and trait anxiety led to a stronger trade-off between pain and performance, in that higher performance led to a greater reduction in pain sensation relative to those lower in pain catastrophizing and trait anxiety. Similarly, for those lower in trait mindfulness, the bidirectional relationship between pain and performance was greater than those higher in mindfulness.

**Table 2:** Standardized coefficients of moderated multilevel mediations.

	a_2_	b_2_	c′_2_	c_2_	ab_2_
**Mediation 1: heat level—sensation—performance (2-back trials alone)**					
Pain catastrophizing	0.000	− 0.183*	0.151*	0.035	− 0.136*
Trait anxiety	0.002	− 0.177*	0.129	0.014	− 0.128^t^
Trait mindfulness	− 0.000	0.226*	− 0.236**	− 0.045	0.177*
**Mediation 2: task difficulty—performance—sensation (pain trials alone)**					
Pain catastrophizing	0.003	− 0.096*	0.010	0.061	0.049*
Trait anxiety	0.014	− 0.102**	0.050	0.118*	0.056*
Trait mindfulness	− 0.008	0.156**	0.038	− 0.053	− 0.084***

Standardized regression coefficients for 2nd-level threat-sensitivity moderators. Coefficients are tested on individual first-level mediations depicted in Fig. 3; ***p < 0.001, **p < 0.01, *p < 0.05, ^t^*p* < 0.07; Mediation path subscript notation previously used elsewhere^21^.

**Figure 4:**
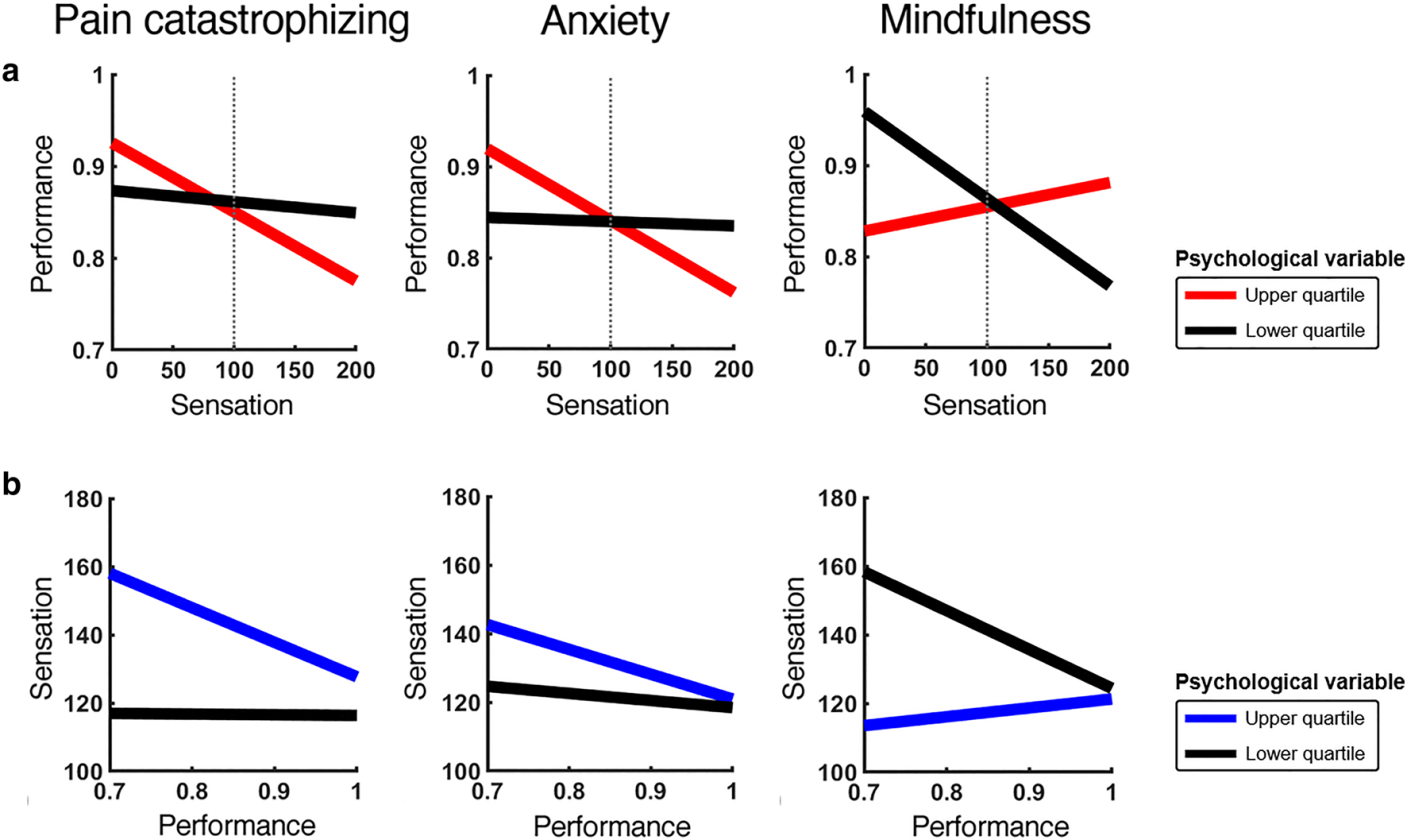
Effects of psychological variables on the trade-off between thermal sensation and performance. Linear functions were estimated using coefficients from the output of the multilevel models displayed in Table 2 (see supplementary material for more information on these analyses). Coloured lines (red, blue) depict the sensation–performance relationship for the upper quartile of participants (n = 10) within the indicated moderator, and black lines depict the lower quartile of participants (n = 10). In red, task performance as a function of sensation in an individual trial (i.e., the *b* path in the pain interference model above), after controlling for the effects of heat level; in blue, sensation as a function of performance (i.e., the *b* path in the task analgesia model above), after controlling for the effects of task difficulty level. Dotted gray vertical lines represent the pain threshold at sensation ratings level 100.

For the task analgesia model, the relationship of performance predicting sensation, after controlling for task difficulty (*b* path), was also significantly positively moderated by trait anxiety and pain catastrophizing, and significantly negatively moderated by trait mindfulness. To further illustrate these moderating effects, we plotted performance as a function of reported sensation in 2-back trials (Fig. 4a, in red), and plotted reported sensation as a function of performance in Pain trials (Fig. 4b, in blue) for the top and bottom quartiles of each moderator (see supplementary material for details on analyses). Briefly, we plotted the average *b* paths for participants from the top and bottom quartiles of scores of each moderating psychological variable for the pain interference and task analgesia models separately. Figure 4a displays the predicted task performance based on ratings of thermal sensation (i.e., pain), after controlling for heat level, during the 2-back task for each of the moderating variables. Figure 4b similarly displays predicted sensation from task performance, after controlling for task difficulty, during high heat (i.e., painful) trials for each of the three moderating variables. Importantly, for both the pain interference and task analgesia models, higher pain catastrophizing, higher trait anxiety, and lower mindfulness predicted a stronger negative relationship between pain and performance (*b* paths in the models). This finding highlights the bidirectional nature of the two processes, in that certain psychological factors can amplify the trade-off between pain and performance, perhaps reflecting differences in how attentional resources are allocated when the two are in competition.

The moderating effects of pain catastrophizing, trait anxiety, and mindfulness on the bidirectional relationship between pain and task performance also influenced the total mediation terms in both of our mediation models, suggesting that all three traits potentiated both the interruptive effects of temperature on performance mediated by increases in pain (Fig. 3a), and the anti-analgesic effects of poorer performance on the more difficult 2-back versus the easier LR task (Fig. 3b). High catastrophizing and low mindfulness also predicted a greater direct effect of heat level on performance (path *c′* in the pain interference mediation model), suggesting that these traits predicted performance-enhancing effects of heat level once controlling for trial-by-trial fluctuations in pain ratings. These seemingly paradoxical effects of heat on performance could reflect performance-enhancing effects of increased vigilance or arousal, as suggested elsewhere^20^. Finally, the only trait showing a significant moderation on total effects (path *c*), was the anti-analgesic effect of trait anxiety on the general analgesic effect of type of task on pain, which suggested that highly anxious individuals experienced less analgesia overall from performing the more difficult task.

## Discussion

According to limited capacity models of attention^4^, pain perception and task performance compete against each other as combined resource requirements exceed working memory capacity. Here, we examined the influence of pain catastrophizing, trait anxiety, and trait mindfulness on how these resources are allocated in the trade-off between pain processing and concurrent task performance. We found a significant effect of these traits, wherein the strength of the bidirectional relationship between pain processing and task performance was separately influenced by each of our psychological variables of interest.

First, using a mediation framework to examine the trial-by-trial relationship between pain and task performance, we found that (1) the reduction of performance at painful heat levels was explained by changes in thermal sensation, and (2) a difficult cognitive task reduces pain perception, but that this analgesic effect was partially suppressed if one failed to maintain good performance of the task. Specifically, when examining the effect of pain interference, we observed that the relationship between heat level and task performance (i.e., how painful heat disrupts task performance) was mediated by subjective thermal sensation on the given trial. After controlling for this mediating effect (*ab* path), we observed no influence of heat level on performance. When examining the effect of task analgesia, we observed that analgesia resulting from engagement in a demanding cognitive task was separately driven by a performance-dependent component and a performance-independent component. That is, we found that higher performance led to a decrease in thermal sensation (*b* path in task analgesia model), but that this effect was suppressed by lower performance at higher task difficulties (*ab* path). After controlling for the effect of performance, we found that engaging in a difficult cognitive task also reduced pain perception (*c′* path). Together, these suggest that simply engaging in a distractive task can reduce one’s perception of pain, and that maintaining good performance enhances this analgesic effect. Most importantly, the inverse relationship between pain and task performance should not be interpreted as one causing the other in a unidirectional manner. Rather, there appears to be a dynamic competition between pain and task performance, wherein the two processes compete for access to a shared limited-capacity pool of a cognitive resources. This study aimed to examine how this competition, and subsequent allocation of resources, was influenced by person-level factors such as pain catastrophizing, trait anxiety, and trait mindfulness.

To address this aim, we extended the mediation models to include our psychological variables as moderators on the different mediation paths. We found that people with higher levels of pain catastrophizing, trait anxiety, and lower levels of trait mindfulness exhibited a stronger bidirectional relationship between pain processing and concurrent task performance (i.e., *b* paths for both models), suggesting that more pain was felt on trials in which they performed poorly, and less pain on trials in which performance was high. By contrast, there appeared to be little to no meaningful trade-off between pain and task performance for people with the opposite trait profile (i.e., darker-coloured slopes in Fig. 4a). However, higher scores of pain catastrophizing, trait anxiety, and lower scores of mindfulness do not necessarily lead to an increase in pain interference or task analgesia per se, but simply suggest that in the trade-off between pain perception and task performance, one can be privileged over the other. That is, for people higher in pain catastrophizing, trait anxiety, or lower in trait mindfulness, processing pain comes at the expense of task performance (and vice versa), while those with the opposite pattern of personality traits (i.e. low catastrophizing, low trait anxiety, and high mindfulness) are better able to appropriately allocate resources between pain perception and task performance. This property could explain the sometimes conflicting reports found in the existing literature; i.e., the balance between pain intensity and task difficulty can vary from person to person, wherein some may experience more pain interference^8–10^, others may experience altered task-induced analgesia^13,22,23^, or neither of the two^24,25^.

Furthermore, in the task analgesia model only the performance-dependent component (i.e., *b* path) appeared to be influenced by threat-related traits. Indeed, poor performance seemed to be more disruptive in high catastrophizers. As mentioned previously, this effect seemed to be driven by the greater bidirectional relationship between pain and performance for high catastrophizers. One particular challenge with n-back tasks is for the participant to remain engaged in the task after having made an error, and it appears that pain could interfere more easily with task performance during these moments of vulnerability in these individuals, thereby further worsening n-back performance and weakening task analgesia. In the case of those with high trait anxiety, this effect was strong enough to have a general impact on analgesia induced by the 2-back task: they exhibited less distraction analgesia, even though they did not report more pain during the control task, did not have greater difficulty with the cognitive task, and were not more sensitive to the thermal stimuli during their calibration. Crucially, this effect of anxiety was revealed only after the task and pain were combined. More specifically, anxious individuals suffered more from the counter-analgesic effect of poor performance on the 2-back task: for them, performing poorly on the 2-back task was associated with more pain—or less analgesia—because of the steeper bidirectional negative relationship between performance and pain. Finally, low levels of mindfulness appear to exert similar effects to those of higher pain catastrophizing and trait anxiety. Higher mindfulness appears to be connected to a wider distributed attentional field^16^ and would suggest that those lower in mindfulness would be less able to balance attentional resources between task performance and pain processing. Indeed, higher mindfulness may promote increased awareness and monitoring of subjective experience without explicit engagement^16^, which may explain the reduced trade-off between pain and performance at higher trait levels. However, the unique role of mindfulness beyond the components shared with pain catastrophizing and anxiety^26^ requires further investigation.

Perhaps surprisingly, a steeper negative relationship between pain and performance also affords the possibility for greater task analgesia in high catastrophizers, as was observed in a previous study^23^. One potential explanation for this counter-intuitive effect is that high catastrophizers may use the concurrent task as an additional motivation to avoid the thermal stimulus by focusing more attention on performing well on the task. This interpretation is consistent with the fear–avoidance model of chronic pain^27^, which builds on current theories of attentional biases in anxiety disorders^28^ that indicate anxious individuals strategically employ attentional avoidance to mitigate their anxiety. More specifically, studies of attentional biases in anxiety disorders typically show that the initial automatic attentional capture observed shortly (500 ms) following the presentation of threat cues is often followed by attentional avoidance (1250 ms after threat cues)^29^. The locus of attention therefore may vary more significantly in anxious individuals, which could explain the greater negative bidirectional relationship observed here between pain perception and task performance.

Our analytical framework may also explain other seemingly counter-intuitive findings previously reported in the literature, such as cognition-enhancing effects of painful temperatures observed in some individuals^20^. Indeed, our findings also revealed significant moderating effects of catastrophizing and mindfulness on the direct effects of temperature on performance once controlling for subjective thermal sensation. In our experiment, this performance-enhancing effect of painful temperatures was overshadowed by an opposite negative effect of temperature on performance mediated through subjective pain ratings, but it is possible that the positive effect may predominate in certain circumstances or in certain individuals. We speculate that these direct cognition-enhancing effects of painful temperatures may be mediated through general effects of pain on sympathetic arousal^30^, which has been shown to induce an optimal state for enhanced flow and performance on executive tasks^31^. In other words, pain is always distracting, but it is also stimulating and therefore under certain circumstances it could increase the total amount of resources to be shared between task performance and pain processing.

The present findings therefore highlight the numerous ways in which psychological factors can interact with each other. Most of these interactions appear dependent on a pivotal trial-by-trial negative relationship between pain and task performance that is responsible for both the disrupting effects of pain on performance and the analgesic effect of task performance on pain. However, as our mediating variables of thermal sensation and performance were not directly manipulated, we caution against inferring direct causality of these effects^32,33^. That is, we emphasize the bidirectional nature of the relationship between pain processing and task performance, more so than a causal link between the two. We found that anxious traits positively related to pain catastrophizing and trait anxiety, and negatively related to mindfulness appear to increase the within-subject trade-off between pain and task performance, likely reflecting increased attentional oscillations between pain and task in anxious individuals that may force the balance to tilt in favor of pain or task performance on any given trial. Our results can therefore provide an integrative framework explaining both common-sense interactions between pain and cognition, such as task analgesia and disruption of cognitive performance by pain, and more counter-intuitive effects such as potentially increased task analgesia in anxious individuals, or cognition-enhancing effects of painful temperatures.

The fact that we did not observe strong disrupting effects of pain on task performance could be due to our particular task design which has the cognitive task begin 7 s prior to the start of the thermal stimulation. Indeed, we can speculate that the temporal precedence of the cognitive task over the painful stimuli may make it easier to prioritize the task because participants may require a few seconds at the start of the 2-back trial to adjust to the task and achieve a state of absorption. Therefore, painful stimuli presented during that early phase of 2-back trials may have a stronger disruptive impact on task performance. Nevertheless, our design mimics the situation where pain interrupts an ongoing task in daily life, which may be highly relevant to understanding pain disability in clinical contexts. Additionally, we used performance as the mediator in our task analgesia mediation model rather than a measure of task engagement such as subjective effort. This was done to reflect our hypothesis of a bidirectional relationship between task performance and pain processing specifically. However, future studies examining mediating effects of task engagement on pain interference and task analgesia should consider the role of subjective effort and concentration in addition to markers of performance.

Other factors in task design may also influence prioritization of pain or the task. For instance, more painful stimuli would be expected to be more distracting. Moreover, a task that is too difficult might lead to disengagement from the task and lower task-related analgesia. Finally, another potentially important factor influencing the trade-off between pain perception and task-related analgesia would be the presence of rewards associated with good task performance, which should be able to boost task performance and associated analgesic effects. Future studies should aim to assess potential interactions between personality traits and these additional factors affecting the trade-off between pain perception and task performance.

Finally, we note that the psychological traits we have chosen to examine in this study are highly correlated with one another, and reflect theoretically similar constructs. Indeed, pain catastrophizers and anxious individuals share a maladaptive cognitive style^34^, and the two constructs typically correlate with one another^35–37^. Moreover, higher mindfulness predicts lower anxiety^38^ and lower pain catastrophizing^19,39,40^. In addition, mindfulness also appears to counteract experiential avoidance^41^, which in our experiment might have translated into a lesser need to suppress attention to pain threat^42^ and thereby a lower trade-off between pain and performance. Despite the correlations between the different constructs, we preferred not to employ a data reduction strategy so as to be better able to link the present findings with the existing literature on these three psychological variables. However, future studies could aim to provide a more exhaustive characterization of psychological factors beyond the threat-related traits that were the focus of our study. Moreover, results from our study could help elucidate the large impact of pain on cognition, and of cognition on pain observed in chronic pain syndromes. Indeed, prolonged exposure to pain may pose a higher burden on attentional systems in anxious individuals, which could fuel a vicious circle leading to the maintenance of pain and disability. If supported by further research, knowledge about the effects of psychological traits on pain-regulating cognitive mechanisms could help provide better guidelines regarding cognitive remediation strategies for patients with chronic pain.

## Methods

### Participants

Fifty-two young adults were recruited from local universities using recruitment posters and advertising on social media websites. Recruitment and data collection took place at the Centre de Recherche de l’Institut Universitaire de Gériatrie de Montréal (CRIUGM). Exclusion criteria included a history of neurological or psychiatric diagnosis, diagnosis of chronic pain syndrome or neuropathy, history of alcohol or substance abuse, and regular (> 2 weekly) use of analgesics, anticonvulsants, narcotics, antidepressants, or anxiolytics. Ethics approval was obtained from the Research ethics committee of the CRIUGM (CER-IUGM 13–14-034). All methods were carried out in accordance with relevant guidelines and regulations. Written informed consent was obtained from all participants, who were all over 18 years of age. All experimentation was completed in English or French.

Fifty-two participants completed the first testing session. Seven participants did not complete the second testing session for the following reasons: one because temperatures intended to be nonpainful (40–44 °C) induced pain at levels intended for the ‘high pain’ condition, one for consistent failure to follow instructions, one for an inability to schedule the second testing session, two because our maximum temperature of 49 °C failed to produce pain, one for failure to tolerate any painful stimuli during sensory calibration at the first visit, and one for software errors that arose near the end of the second session. As a result, 45 participants therefore completed both testing sessions. After data collection was complete, we excluded data from four more participants due to the following: one for significant (> 50%) missing data, two for inability to tolerate more than 50% of painful behavioral trials, and one because their 2-back performance was near chance for the second testing session of the experiment.

### Design

We designed a paradigm in which we combined two levels of thermal stimulation (non-painful warm, painful heat) with two levels of cognitive task engagement (simple left–right identification control task, 2-back working memory task). We chose the 2-back task because it engages executive functions^43^ and requires continuous performance. We pre-calibrated the thermal stimuli to ensure equivalent warm and pain levels across participants, and also pre-calibrated the difficulty to ensure equivalent levels of performance across participants. Our aim was to verify whether analgesic effects of the 2-back task were mediated by performance on the task, and conversely, to test whether any task-interruptive effects of the painful stimulus were mediated by reported pain. Finally, we used questionnaire measures of trait anxiety, pain catastrophizing, and trait mindfulness in our multilevel mediation analyses to determine their moderating effects on the first-order mediations.

### General procedure

In order to prevent cognitive fatigue induced by the calibration task from affecting performance in the main experimental task, we chose to separate them across two sessions. In session one, after providing informed consent, participants completed the Five-Facet Mindfulness Questionnaire followed by the sensory calibration procedure (Fig. 1a). In session two, one to ten days later, participants completed the Pain Catastrophizing Scale, the State-Trait Anxiety Inventory., and the 2-back task calibration procedure (Fig. 1b). Lastly, they completed the main experimental task, in which they completed blocks of the pre-calibrated 2-back or left–right discrimination control task while receiving the individualized thermal pain or warm stimuli (Fig. 1b,c). Details on the individual steps are provided below.

### Psychological measures

#### Moderator 1: pain catastrophizing scale

Pain catastrophizing is the tendency to engage in excessive negative elaborations about pain, to magnify or exaggerate its threat value, and to feel helpless in the face of pain^44^. It has received extensive support as a predictor of pain and associated disability^45^. The Pain Catastrophizing Scale (PCS) is a 13-item measure comprised of three subscales: rumination, magnification, helplessness. The PCS and its French-Canadian adaptation have demonstrated good psychometric properties^46,47^. PCS scores have been found to have adequate internal consistency (α = 0.87), high construct validity^48^ and moderate test–retest reliability (r_XX_ = 0.67)^49^. In our experiment, our questionnaire instructions elicited attitudes towards pain *in general* rather than specifically towards thermal pain induced during our study, and as such, we probed *trait* pain catastrophizing.

#### Moderator 2: state-trait anxiety inventory—trait subscale

Trait anxiety is the relatively stable tendency to experience excessive worry and tension, and increased autonomic reactivity to psychological stressors, and those with high trait anxiety scores tend to experience more situations as threatening or dangerous^50^. The STAI has demonstrated high internal consistency (average α > 0.89)^51^, and the Trait portion of Spielberger's State-Trait Anxiety Inventory is a 20-item questionnaire with high test–retest reliability (r_XX_ = 0.88)^51^ and high validity ^52^ with respect to clinical ratings and other anxiety questionnaires. It also has good psychometric properties in French^53^. We selected the Trait portion only for analyses since it is a good predictor of pain-related fear traits^45^.

#### Moderator 3: five facet mindfulness questionnaire

Trait mindfulness is associated with an open awareness and non-judgmental acceptance of one's emotions, thoughts and sensations on a moment-to-moment basis^16^. The 39-item five facet mindfulness questionnaire (FFMQ) comprises five dimensions: observing, acting with awareness, describing, non-judgment, and non-reacting^54^. The FFMQ has good construct validity in a non-meditating sample^55^, adequate to good test–retest reliability (r_xx_ = 0.657 to 0.863)^56^, and good internal consistency (α = 0.86 to 0.93)^57^.The French version also has good psychometric properties^58^. A FFMQ 'global mindfulness' sum score was calculated leaving out the ‘observing’ facet, which is the least correlated with other facets in non-expert meditators^59^. Hence, we use *4-FFMQ* throughout the text to reflect this decision to exclude the ‘observing’ sub-scale from the average score.

### Sensory calibration and ratings

We completed a sensory calibration procedure for each participant in order to control for the effects of individual differences in pain sensitivity during the pain-task procedure^60^. The on-screen sensory evaluation scales were presented with the E-prime software package version 2.0 (Psychology Software Tools, Inc., 2002, accessible at www.pstnet.com), and an experimenter delivered all thermal stimulations with a Medoc Thermode 9 cm^2^ contact probe (TSA Neuro-sensory analyzer, Medoc Ltd. Advanced Medical System, Israel).

Prior to the calibration procedure, the experimenter instructed the participant to apply the thermode to the fingers or palm of their dominant hand and were given three familiarization stimulations at 40, 44 and 46 °C for 5 s each. When these were complete, the experimenter provided instructions on how to complete the sensory ratings on-screen as follows: ‘After a stimulation, you will see a screen saying either “not painful” on the left or “painful” on the right. You must left-click if it was not painful and right-click if it was painful.’ If the participant left-clicked ‘not painful’, the next screen displayed a horizontal visual analogue scale (VAS^61^, also see^62^) with the anchors 'no warmth at all' on the left and 'very hot, without pain' on the right and participants were instructed to use the mouse to slide the on-screen cursor to the place on the scale that best corresponded to their perception. A corresponding numerical rating from 0 to 100 was displayed in real-time underneath the VAS. If participants right-clicked ‘painful’, they were first presented with a horizontal VAS on which to record pain intensity (described to participants as ‘the strength of the sensation’), with left and right anchors ‘not intense at all' and 'extremely intense’ and a numerical rating from 0 to 100 corresponding to the position of the cursor on the VAS; this was immediately followed by presentation of a VAS for pain unpleasantness (described as ‘the degree to which the pain was bothersome or uncomfortable’) with left and right anchors 'not unpleasant at all' and 'extremely unpleasant'. Participants were provided unlimited time to practice using the sensory rating interface.

We selected the electronic VAS combined with a corresponding numerical rating scale (NRS), because they have been shown to be useful and effective for rapid unidimensional measurement of pain. In addition, the VAS has been shown to have ratio scale characteristics, which allows us to fit a stimulus–response curve based on a power function to calculate target thermal stimulation temperatures for our sensory calibration^63^. An overview of psychometric properties of different rating scales^64^ found that the NRS has been demonstrated to have moderate test–retest reliability in clinical samples (between 0.67 and 0.96)^65,66^. The NRS and VAS have a convergent validity of 0.79–0.95, suggesting that they measure the same construct and yield similar results. For the VAS, test–retest reliability has been shown to be high (0.71–0.99^65,66^); and for the electronic VAS, it is even higher (0.88–0.99)^67^. Our digital version of the VAS also circumvents experimenter measurement error inherent in a mechanical or paper–pencil scale. However, some limitations in the VAS rating method have been noted. A systematic review found that the NRS was found to be easier to use and had better compliance than the VAS across clinical samples^68^. Another systematic review found that the VAS was less practical than alternate rating methods in the elderly and in those with cognitive impairments, and recommended its combination with another appropriate scales (e.g. the NRS)^69^. We therefore hoped to mitigate shortcomings of the NRS and the VAS by combining them. Our combined method is very similar to that used in a study that showed that it was able to discriminate ratings related to small (0.5 °C) temperature increments, similarly to VAS and NRS alone, that adding a numerical scale to the VAS did not appear to change its measurement properties and that the VAS and numerical rating together was preferred as a rating method over the VAS or the NRS alone^70^.

Following practice, participants were instructed to use the rating scales to evaluate a series of thermal stimuli. Calibration consisted of the application of a series of 28 stimulations administered to the volar surface of the non-dominant arm. Seven temperatures (40, 44, 45, 46, 47, 48, and 49 °C) were presented, one to each of four sites, the order of which was determined using a pseudorandom Latin square design. Four different testing orders were used across the subjects. Heat was applied for a total of 13 s (2.5 s rise time from 32 °C baseline, 8 s plateau, 2.5 s fall time to baseline), during which participants were presented an on-screen fixation cross; they then were prompted to rate the sensation as practiced previously. The sensory calibration procedure lasted approximately 20 min.

While this way of collecting pain ratings allows the evaluation of pain separately from non-painful sensations, we decided to combine non-painful warmth and pain intensity on the same continuum (warmth: 0–100; pain 100–200), so as to prevent potential flooring effects when thermal sensations are reported to be below the pain threshold^71,72^. Finally, because pain unpleasantness has a clearly affective component and non-painful warmth does not, it could not theoretically be combined with non-painful warmth perception on the same scale. We therefore preferred to restrict our analyses to the combined warmth—pain intensity ratings.

Sensory intensity ratings were corrected for presentation order and stimulation site effects. Pain sensitization and habituation resulting from peripheral or central adaptation processes can occur with repeated painful stimulations, and can be site-specific and site-nonspecific^73^. We therefore corrected the raw pain ratings for these processes using Jepma et al.’s^73^ dynamic model, which we computed in Matlab 2012a. We then plotted all corrected sensation intensity reports as a function of stimulation temperature. Exponential curve fitting using a Matlab function permitted selection of a Warm (80/100 of warmth) and Pain (40/100 of pain) temperature for each participant. The maximal temperature of 49 °C was used for three participants for whom the predicted pain temperatures surpassed 49 °C (approximate predicted 40/100 pain temperatures of 49.7 °C, 49.6 °C, and 49.2 °C; predicted pain ratings at 49 °C of 15/100, 20/100, and 35/100, respectively). Including or removing these three participants did not change the results of our analyses.

### Cognitive tasks

#### 2-back task

The 2-back task was programmed with E-Prime software package, from a script adapted from Buhle and Wager^3^. Participants were instructed on the task by the experimenter as follows: ‘You will see a series of letters appear on the screen. For each letter, you must left-click if the letter is the *same* as the letter seen two steps previously, and right-click if the letter is *different*. Each letter requires a response.’ Each trial lasted 20 s and featured a series of items, where each item presentation consisted of a fixation cross (250 ms), followed by a letter (500 ms), followed by a blank interval. Responses were recorded only in the form of mouse-clicks during letter presentation and the blank interval. Possible letters used were C, F, J, N, Q, S, V, and X. In all trials, on average 25% of letters (excluding the first two) were targets. For our specific task, longer intervals increased task performance, an effect that we attribute to increased time allocated for rehearsal of the letters in working memory. Since our aim was to recruit working memory, we were careful to discourage responses based on familiarity alone by including lures (1-back or 3-back occurrences) on 12.5% of letters. The same 2-back working memory task was used during the calibration procedure as during the behavioral pain-task procedure.

#### Performance calculation

For each trial, performance accuracy was calculated as A, a non-parametric signal detection measure^74^. In signal detection theory, A is calculated as an estimation of the approximate area under the receiver operating characteristic (ROC) curve of the graphical plot illustrating the ability of a binary classifier system in which the discrimination threshold is varied. It is an alternative to the sensitivity index d’, calculated as hits – false alarm rate under the assumption that target and noise are normally distributed. In our study, the curve represents the proportion of correct detections of 2-back targets as a function of false positives, where the area under the curve represents the proportion of correctly obtained responses. A is equal to 0.50 at chance, and 1.00 at perfect performance, and, unlike d’, is calculated as an approximate ‘average’ of the minimum-area and maximum-area proper ROC curves, and does not depend on distributional assumptions of the underlying data. Task performance calculation was identical in the 2-back calibration procedure and in the main pain-task procedure.

#### Task calibration

Participants were provided instruction on the task, followed by unlimited 2-back practice trials with computerized performance feedback until they felt comfortable with the task. They were then instructed as follows: “You are now going to perform the 2-back task several times. Sometimes the letters will go faster. If the task becomes difficult, just do your best, and trust your intuition. If you are not sure what respond, just guess; but make sure that you provide a response for every letter.” Participants completed 18 2-back trials without performance feedback. Following a procedure used previously^3^, task performance on the previous trial was calculated and the blank interval duration after each letter was manipulated in a staircase procedure to maintain a task performance of 0.75 < A < 0.85. The starting blank interval duration was 2583 ms. If performance on two subsequent trials was above A = 0.85, the blank interval duration was decreased for the following trial. If performance on two subsequent trials was below A = 0.75, interval duration was increased for the following trial. In order to maintain constant total trial duration of 20 s, faster trials contained more items (range 6 to 26 letters). At the end of the calibration procedure, the final blank interval duration was derived as the participant's task speed parameter. This was done to ensure that the task remained equally challenging and engaging across participants. The shortest and longest achievable intervals were 19 ms and 2583 ms, respectively. The shortest interval attained across the whole sample was 159 ms (22 letters), and the longest was 1750 ms (8 letters), with an average of 579 ms (SD = 346 ms).

#### Control task

After having completed the 2-back calibration procedure, participants received instructions on how to complete the control task as follows: ‘You will see a series of left- and right-pointing arrows on the screen. For each arrow, you must left-click for left-pointing arrows or right-click for right-pointing arrows.’ Participants were given unlimited practice trials until they were comfortable with the task. As with the 2-back task, each trial lasted 20 s and featured a variable number of items, each consisting of a fixation cross (250 ms), followed by an arrow (500 ms), followed by a blank interval. Participant’s blank interval duration was set to the same one derived from their 2-back performance calibration procedure. The proportion of left-pointing arrows was the same as that of targets in the 2-back task.

### Behavioral assessment of the trade-off between pain and task

The design of the pain-task procedure crossed both cognitive tasks and both heat levels in a within-subjects design, resulting in four conditions with nine trials of each type, for a total of 36 trials. These were presented in a pseudorandom order, which was the same for each participant. Participants were instructed as follows: “You are now going to have to complete the 2-back or the left–right task, in combination with some heat applied to your arm. At the end of the stimulation and the task, you will have to evaluate the warmth or pain, just as you did in your first visit. Here's a reminder of how it's done.” Participants were then given a reminder of the use of the digital VAS. They were instructed to do their best on the cognitive tasks, although they were given no instruction regarding prioritization of the task or stimulus.

A trial involved completing either the 2-back or the control task while receiving either a warm or painful thermal stimulus; immediately following the end of the task and concurrent pain, the participant rated their sensation using rating scales identical to those used in the sensory calibration procedure (Fig. 1c). Trials lasted between 45 and 60 s each, depending on how participants rated the thermal stimulus (warmth rating involved a single VAS, while pain rating involved two VAS). The pain–task procedure lasted approximately 30 min.

### Analyses

Pain ratings from the behavioral procedure were subjected to the same correction procedure applied to sensory calibration ratings to account for any habituation or skin site-related differences in sensitivity during the experimental conditions^73^. For all analyses, pain and performance measures were z-transformed within participants to account for further inter-individual differences in pain perception and task performance.

In order to examine trial-by-trial interactions between task performance and pain, we performed multilevel mediation analyses^21^ on all behavioural trials as performed previously^3^ (see Supplementary Methods). A mediation analysis tests whether the covariance between a predictor (*X*) and a dependent variable (*Y*) can be explained by a third variable, the mediator (*M*)^75,76^. The mediation analyses were performed using 'mediation.m' custom Matlab script, available online (https://github.com/canlab/MediationToolbox/tree/master/mediation_toolbox). We tested the significance of the ab mediation paths^77^ with a bias-corrected bootstrap test^78,79^ with 10,000 bootstrap samples to test each of the *a*, *b*, c, *c′*, and *ab* path coefficients. Testing time interval in days, pain threshold, and task calibration interval were entered as covariates from each moderator prior to running the general linear models. In all tests, alpha level for significance was set to 0.05. First, we tested whether reported sensation mediates the effect of stimulation level on task performance, in 2-back trials alone. Next, we tested whether task performance mediated analgesic effects of task difficulty on reported sensation, in pain trials alone. Of particular interest to us were the first-level *b* paths in both models, which represent the relationship between reported sensation and of task performance on each other.

In order to test our hypotheses about the effects of interindividual differences on outcomes in a pain-task paradigm, second-level moderators, in particular pain catastrophizing, trait anxiety (STAI-T), and mindfulness, were each separately tested as second-level moderators of first-level effects. Finally, the interval between testing sessions, individual pain thresholds, and task calibration intervals were covaried out for each moderator prior to running the general linear models to ensure that moderator effects could not be explained by these variables, even though none of the covariates correlated significantly with any of the moderators (see Table 1).

## Supplementary Information

Supplementary Information.

## Data Availability

The datasets analysed during the current study are available from the corresponding author on reasonable request.
