# Supplementary material for: Inter-individual predictors of pain inhibition during performance of a competing cognitive task
Source: Sci Rep. 2020 Dec 11;10:21785. doi: 10.1038/s41598-020-78653-z (PMC7732830; doi:10.1038/s41598-020-78653-z)
Supplement: Supplementary file 1 — Supplementary Information. [file 41598_2020_78653_MOESM1_ESM.docx]

**Supplementary material for: Inter-individual predictors of pain inhibition during performance of a competing cognitive task**

V. Tabry ^a^, T. A. Vogel ^b^, M. Lussier ^cd^, P. Brouillard ^e^, J. Buhle ^f^, P. Rainville ^cg^, L. Bherer ^chi^, M. Roy ^bcj^

**Complete affiliations of authors**

a Faculty of Medicine, McGill University, Canada

b Department of Psychology, McGill University, Canada

c Centre de Recherche de l'Institut Universitaire de Gériatrie de Montréal (CRIUGM), Canada

d Faculté de Médecine, Université de Montréal, Canada

e Mila, Université de Montréal, Canada

f University of Southern California, United States of America

g Département de stomatologie, Faculté de Médecine Dentaire, Université de Montréal, Canada

h Montreal Heart Institute, Canada

i Département de Medicine, Université de Montréal, Canada

j Alan Edwards Center for Research on Pain (AECRP), McGill University, Canada

**Corresponding author:**

Mathieu Roy

Department of Psychology, McGill University

Suite 3100, Genome Building,

740 Dr. Penfield Avenue

Montreal, QC, Canada H3A 0G1

Tel: (514)398-4234

e-mail: mathieu.roy3@mcgill.ca

**Supplementary Table 1**

*Review of pain interference and task analgesia literature in pain-free adults from 1990 to 2019*

|  | |  |  | Stimulus | |  | Task | |  |  |  |  |
| --- | --- | --- | --- | --- | --- | --- | --- | --- | --- | --- | --- | --- |
| Author & date | | Exp | N | Type | Calib |  | Type | Calib | PI | TA | Mod | Mod effects |
| Attridge et al. 2016 ^1^ |  | | 44 (22F) | Heat | Yes |  | Switching task | No | No | - | - |  |
| Bantick et al. 2002 ^2^ |  | | 8 (2F) | Heat | Yes |  | Counting Stroop | No | - | Yes | - |  |
| Bingel et al. 2007 ^3^ | |  | 16M | Laser | No |  | n-back | No | Yes | Yes | - |  |
| Boselie et al. 2014 ^4^ | |  | 74(58F) | CPT | No |  | operation span task | No | Yes | - | Yes | Induced optimism abolishes pain-related task interference |
| Boselie et al. 2016 ^5^ | | 1 | 61 (45F) | CPT | No |  | 2-back task | No | No | - | - |  |
|  | | 2 |  | Heat | Yes |  | 2-back task | No | Yes | - | No | Induced optimism did not protect from pain-related task interruption |
| Brooks et al. 2002 ^6^ | |  | 18 (6F) | Heat | Yes |  | Global motion discrimination | No | - | - | - |  |
| Buhle & Wager 2010 ^7^ | |  | 24 (15F) | Heat | Yes |  | 3-back | Yes | Yes | Yes | - |  |
| Buhle et al. 2012 ^8^ | |  | 33 (19F) | Heat | Yes |  | 3-back | No | - | Yes |  |  |
| Campbell et al. 2010 ^9^ | |  | 32 (15F) | Capsaicin + Heat | No |  | Video Games | No | - | Yes | Yes | Catastrophizing delayed the onset of task analgesia |
| Coen et al. 2008 ^10^ | |  | 12M | Esophageal pressure | Yes |  | 1-back | No | No | Yes | - |  |
| Crombez et al. 1994 ^11^ | |  | 44 (33F) | Radiant Heat | No |  | Tone discrimination | No | No | - | Yes | Warnings about incoming stimuli reduced SCR and reported pain |
| Crombez et al. 1996 ^12^ | |  | 26 (16F) | ECS | Yes |  | Tone discrimination | No | Yes | - | - |  |
| Crombez et al. 1997 ^13^ | |  | 24 (15F) | ECS | Yes |  | Tone discrimination | No | Yes | - | - |  |
| Crombez et al. 1998a ^14^ | |  | 38 (16F) | ECS | No |  | Tone discrimination | No | Yes | - | Yes | High-threat instructions increased interference |
| Crombez et al. 1998b ^15^ | | 1 | 44 (32F) | ECS | No |  | Tone discrimination | No | No | - | Yes | Catastrophzing increased interference and increased effect of threat instructions on task performance |
|  | | 2 | 36 (26F) | ECS | No |  | Tone discrimination | No | No |  | Yes | Catastrophzing increased interference |
| Crombez et al. 2002 ^16^ | |  | 67 (48F) | ECS | No |  | Tone discrimination | No | Yes | - | Yes | Catastrophizing but not negative affectivity increased PI |
| Dick et al. 2006 ^17^ | |  | 16 (11F) | Ischemic pain | No |  | Auditory mismatch negativity | No | No | - | - | - |
| Dowman 2004 ^18^ | |  | 28 (5F) | TENS sural nerve | Yes |  | Subtraction | No | - | Yes | - | - |
| Erpelding et al. 2013 ^19^ | |  | 80 (40F) | Tonic Heat | Yes |  | Numerical interference | No | No | No | - | - |
| Frankenstein et al. 2001 ^20^ | |  | 12 (6F) | CPT | Yes |  | Verbal attention | No | - | Yes | - | - |
| Hodes et al. 1990 ^21^ | |  | 45 (29F) | CPT | No |  | Mental arithmetic | No | - | Yes | - | - |
| Hood et al. 2013 ^22^ | |  | 78 (39F) | CPT | No |  | Letter-number sequencing | No | MIXED | - | - | - |
| Houlihan et al. 2004 ^23^ | |  | 21(9F) | CPT | No |  | Sternberg | No | MIXED | No | - | - |
| Keogh et al. 2013 ^24^ | | 1 | 62 (40F) | Heat | Yes |  | Breakfast-making (planning & multitasking) | No | Yes | - | - | - |
|  | | 2 | 62 (40F) | Heat | Yes |  | word generation (planning & multitasking) | No | No | - | No | Catastrophizing did not increase PI |
| Kóbor et al. 2009 ^25^ | |  | 15 (5F) | Capsaicin + pinprick | No |  | Facial discrimination | No | - | Yes | - | - |
| Lautenbacher et al. 2007 ^26^ | |  | 40 (20F) | Heat + ECS | Yes |  | Counting | No | - | Yes | - | - |
| Larsen et al. 2019 ^27^ | |  | 28 (15F) | Hypertonic saline injection | No |  | 2-back task | No | - | No | - | Task increased pain |
| Moore et al. 2012 ^28^ | | 1 | 20 (13F) | Heat | Yes |  | Sustained attention | No | No | - | - | - |
|  | | 2 | 20 (10F) | Heat | Yes |  | Flanker | No | No | - | - | - |
|  | | 3 | 20 (16F) | Heat | Yes |  | Posner | No | No | - | - | - |
|  | | 4 | 20 (14F) | Heat | Yes |  | 2-back | No | Yes | - | - | - |
|  | | 5 | 20 (14F) | Heat | Yes |  | Go/No-go | No | No | - | - | - |
|  | | 6 | 20 (7F) | Heat | Yes |  | Switch (prime) | No | Yes | - | - | - |
|  | | 7 | 20 (12F) | Heat | Yes |  | Dual-task | No | Yes | - | - | - |
| Moore et al. 2013 ^29^ | | 1 | 50 (29F) | Heat | Yes |  | N-back | No | Yes | - | No | High-threat instructions had no effect |
|  | | 2 | (w/in Subjects) | Heat | Yes |  | Switching task | No | Yes | - | No | High-threat instructions had no effect |
|  | | 3 | (w/in Subjects) | Heat | Yes |  | Divided attention | No | No | - | No | High-threat instructions had no effect |
| Moore et al. 2017 ^30^ | |  | 55 (36F) | Heat | Yes |  | Edinburgh Virtual Errands Task | No | No | - | Yes | Those who self-reported greater pain-related cognitive interruption in daily life had worse performance than those with less self-reported interruption |
| Paris et al. 2013 ^31^ | |  | 38 (19F) | Heat | Yes |  | 2-back task | No | - | No | - | - |
| Petrovic et al. 2000 ^32^ | |  | 10M | CPT | No |  | Computer maze | No | No | Yes | - | - |
| Pud et al. 2008 ^33^ | |  | 60 (46F) | Heat | No |  | Tone discrimination | No | No | Yes | - | - |
| Raudenbush et al. 2009 ^34^ | | 1 | 30 (22F) | CPT | No |  | Videogames | No | - | Yes | No | Aggressiveness and competitiveness had no effect |
|  | | 2 | 27 (13F) | CPT | No |  | Videogames (sports and fighting types) | No | - | Yes | No | Aggressiveness and competitiveness had no effect |
| Remy et al. 2003 ^35^ | |  | 12 (6F) | Heat | Yes |  | Word generation | No | - | Yes | - | - |
| Roelofs et al. 2004 ^36^ | |  | 90F | CPT | No |  | Tone discrimination | No | - | No | Yes | TA observed in low fear of pain group; pain increase observed in high fear of pain group |
| Schlereth et al. 2003 ^37^ | |  | 10 (6F) | Laser | No |  | Subtraction | No | - | Yes | - | - |
| Schrooten et al. 2013 ^38^ | |  | 98 (76F) | ECS | No |  | Impression formation (perseveration task) | No | No | - | Yes | Pain catastrophizing reduced time allocated to task during pain |
| Seminowicz et al. 2004 ^39^ | |  | 18 (10F) | TENS median nerve | Yes |  | Counting Stroop | No | No | - | - | - |
| Seminowicz & Davis 2007 ^40^ | |  | 23 (12F) | TENS median nerve | Yes |  | Multisource interference | No | No | No | - | - |
| Tabry et al. | |  | 41 (21F) | Heat | Yes |  | 2-back | Yes | No | Yes | Yes | Pain catastrophizing, trait anxiety, and low mindfulness, increased the tradeoff between task performance and pain, only anxiety decreased mean TA |
| Slepian et al. 2017 ^41^ | |  | 105 (61F) | Heat | Yes |  | Mental arithmetic | No | Yes | - | Yes | Resilience increased task persistence and performance during pain |
| Stancak et al. 2017 ^42^ | |  | 24 (12F) | Laser | Yes |  | Rubin Face-Vase illusion task | No | - | No | - |  |
| Terkelsen et al. 2004 ^43^ | |  | 26M | TENS sural nerve | Yes |  | Mental arithmetic | No | - | Yes | - | - |
| Valet et al. 2004 ^44^ | |  | 7 (1F) | Heat | Yes |  | Stroop | No | - | Yes | - | - |
| Van Damme et al. 2004 ^45^ | |  | 37 (31F) | ECS | No |  | Tone discrimination | No | Yes | - | - | - |
| Van Damme et al. 2008 ^46^ | |  | 101(79F) | CPT | No |  | Random interval repetition | No | No | Yes | Yes | High-threat instructions increased PI, did Not affect TA |
| Van Ryckeghem et al. 2012a ^47^ | |  | 60 (48F) | ECS | Yes |  | Task-switching paradigm | No | Yes | - | - | - |
| Van Ryckeghem et al. 2012b ^48^ | |  | 53 (42F) | ECS | No |  | Dot-probe task | No | - | Yes | Yes | High attentional bias toward pain reduced TA; pain catastrophizing and state-trait anxiety had no effect |
| Van Ryckeghem et al. 2018 ^49^ | |  | 58 (47F) | CPT | No |  | Random interval repetition | No | Yes | - | No | One session of attention bias modification training did Not reduce the effect of pain on task performance |
| Vancleef & Peters 2006^† 50^ | |  | 48 (36F) | ECS | No |  | Auditory tone discrimination | No | Yes | - | Yes | Pain catastrophizing increased PI |
| Veldhuijzen et al. 2006 ^51^ | | 1 | 16 (8F) | CPT | No |  | Visual search | No | No | - | - | - |
|  | | 2 | 14M | CPT | No |  | Visual search | No | No | Yes | - | - |
| Verhoeven et al. 2011 ^52^ | |  | 91 (72F) | CPT | No |  | Random interval repetition | No | - | Yes | Yes | Cognitive inhibition, but Not switching, predicted faster responses on task during CPT (No pain-free comparator condition) |
| Verhoeven et al. 2012 ^53^ | |  | 87 (44F) | CPT | No |  | Random interval repetition | No | - | No | Yes | Pain catastrophizing increased pain during distraction relative to control |
| Vuong et al. 2018 ^54^ | | 1 | 22 (13F) | Pressure to fingertip | Yes |  | 3-back task | No | - | Yes | - | An n-back task, compared to No task, increased the force required to achieve 5/10 of pain, with no difference between 1- and 3-back tasks |
|  | | 2 | 31 (23F) | Pressure to fingertip | Yes |  | Visual search task | No | - | Yes | Yes | Pain attenuation by a visual search task was increased by high-threat instructions |
| Wiech et al. 2005 ^55^ | | 1 | 11 (3F) | Capsaicin & Heat | Yes |  | Rapid serial visual processing | No | No | - | - | - |
|  | | 2 | 15 (5F) | Capsaicin & Heat | Yes |  | Rapid serial visual processing | No | - | Yes | - | - |
| Yamasaki et al. 2000 ^56^ | |  | 11 (3F) | ECS | Yes |  | Mental calculation or memorization | No | - | Yes | - | - |

**References for supplementary table 1**

1. Attridge, N., Keogh, E. & Eccleston, C. The effect of pain on task switching: Pain reduces accuracy and increases reaction times across multiple switching paradigms. *Pain* **157**, 2179–2193 (2016).

2. Bantick, S. J. *et al.* Imaging how attention modulates pain in humans using functional MRI. *Brain* **125**, 310–319 (2002).

3. Bingel, U., Rose, M., Gläscher, J. & Büchel, C. fMRI Reveals How Pain Modulates Visual Object Processing in the Ventral Visual Stream. *Neuron* **55**, 157–167 (2007).

4. Boselie, J. J. L. M., Vancleef, L. M. G., Smeets, T. & Peters, M. L. Increasing optimism abolishes pain-induced impairments in executive task performance. *Pain* **155**, 334–340 (2014).

5. Boselie, J. J. L. M., Vancleef, L. M. G. & Peters, M. L. The effects of experimental pain and induced optimism on working memory task performance. *Scandinavian Journal of Pain* **12**, 25–32 (2016).

6. Brooks, J. C. W., Nurmikko, T. J., Bimson, W. E., Singh, K. D. & Roberts, N. fMRI of Thermal Pain: Effects of Stimulus Laterality and Attention. *NeuroImage* **15**, 293–301 (2002).

7. Buhle, J. T. & Wager, T. D. Performance-dependent inhibition of pain by an executive working memory task. *Pain* **149**, 19–26 (2010).

8. Buhle, J. T., Stevens, B. L., Friedman, J. J. & Wager, T. D. Distraction and Placebo: Two Separate Routes to Pain Control. *Psychological Science* **23**, 246–253 (2012).

9. Campbell, C. M. *et al.* Catastrophizing delays the analgesic effect of distraction. *Pain* **149**, 202–207 (2010).

10. Coen, S. J. *et al.* Effects of attention on visceral stimulus intensity encoding in the male human brain. *Gastroenterology* **135**, 2065–2074 (2008).

11. Crombez, G., Baeyens, F. & Eelen, P. Sensory and temporal information about impending pain: The influence of predictability on pain. *Behaviour Research and Therapy* **32**, 611–622 (1994).

12. Crombez, G., Eccleston, C., Baeyens, F. & Eelen, P. Disruptive nature of pain: An experimental investigation. *Behaviour Research and Therapy* **34**, 911–918 (1996).

13. Crombez, G., Eccleston, C., Baeyens, F. & Eelen, P. Habituation and the interference of pain with task performance. *Pain* **70**, 149–154 (1997).

14. Crombez, G., Eccleston, C., Baeyens, F. & Eelen, P. Attentional disruption is enhanced by the threat of pain. *Behaviour Research and Therapy* **36**, 195–204 (1998).

15. Crombez, G., Eccleston, C., Baeyens, F. & Eelen, P. When somatic information threatens, catastrophic thinking enhances attentional interference. *Pain* **75**, 187–198 (1998).

16. Crombez, G., Eccleston, C., Van Den Broeck, A., Van Houdenhove, B. & Goubert, L. The effects of catastrophic thinking about pain on attentional interference by pain: No mediation of negative affectivity in healthy volunteers and in patients with low back pain. *Pain Research and Management* **7**, 31–39 (2002).

17. Dick, B. D. *et al.* Effects of experimentally induced pain on mismatch negativity. *Journal of Psychophysiology* **20**, 21–31 (2006).

18. Dowman, R. Distraction produces an increase in pain-evoked anterior cingulate activity. *Psychophysiology* **41**, 613–624 (2004).

19. Erpelding, N. & Davis, K. D. Neural underpinnings of behavioural strategies that prioritize either cognitive task performance or pain. *Pain* **154**, 2060–2071 (2013).

20. Frankenstein, U. N., Richter, W., McIntyre, M. C. & Rémy, F. Distraction modulates anterior cingulate gyrus activations during the cold pressor test. *NeuroImage* **14**, 827–836 (2001).

21. Hodes, R. L., Rowland, E. W., Lightfoot, N. & Cleeland, C. S. The effects of distraction on responses to cold pressor pain. *Pain* **41**, 109–114 (1990).

22. Hood, A., Pulvers, K. & Spady, T. J. Timing and gender determine if acute pain impairs working memory performance. *Journal of Pain* **14**, 1320–1329 (2013).

23. Houlihan, M. E. *et al.* Assessing the effect of pain on demands for attentional resources using ERPs. *International Journal of Psychophysiology* **51**, 181–187 (2004).

24. Keogh, E., Moore, D. J., Duggan, G. B., Payne, S. J. & Eccleston, C. The disruptive effects of pain on complex cognitive performance and executive control. *PLoS ONE* **8**, 10.1371/journal.pone.0083272 (2013).

25. Kobor, I., Gul, V. & Vidnyonszky, Z. Attentional modulation of perceived pain intensity in capsaicin-induced secondary hyperalgesia. *Experimental Brain Research* **195**, 467–472 (2009).

26. Lautenbacher, S., Prager, M. & Rollman, G. B. Pain additivity, diffuse noxious inhibitory controls, and attention: a functional measurement analysis. *Somatosensory & motor research* **24**, 189–201 (2007).

27. Larsen, D. B. *et al.* Corticomotor excitability reduction induced by experimental pain remains unaffected by performing a working memory task as compared to staying at rest. *Experimental Brain Research* **237**, 2205–2215 (2019).

28. Moore, D. J., Keogh, E. & Eccleston, C. The interruptive effect of pain on attention. *The Quarterly Journal of Experimental Psychology* **65**, 565–586 (2012).

29. Moore, D. J., Keogh, E. & Eccleston, C. The effect of threat on attentional interruption by pain. *Pain* **154**, 82–88 (2013).

30. Moore, D. J. & Law, A. S. The disruptive effects of pain on multitasking in a virtual errands task. *Scandinavian Journal of Pain* **16**, 29–35 (2017).

31. Paris, T. A., Misra, G., Archer, D. B. & Coombes, S. A. Effects of a force production task and a working memory task on pain perception. *Journal of Pain* **14**, 1492–1501 (2013).

32. Petrovic, P., Petersson, K. M., Ghatan, P. H., Stone-Elander, S. & Ingvar, M. Pain-related cerebral activation is altered by a distracting cognitive task. *Pain* **85**, 19–30 (2000).

33. Weissman-Fogel, I., Sprecher, E. & Pud, D. Effects of catastrophizing on pain perception and pain modulation. *Experimental Brain Research* **186**, 79–85 (2008).

34. Raudenbush, B., Koon, J., Cessna, T. & McCombs, K. Effects of playing video games on pain response during a cold pressor task. *Perceptual and motor skills* **108**, 439–448 (2009).

35. Rémy, F., Frankenstein, U. N., Mincic, A., Tomanek, B. & Stroman, P. W. Pain modulates cerebral activity during cognitive performance. *NeuroImage* **19**, 655–664 (2003).

36. Roelofs, J., Peters, M. L., Van Der Zijden, M. & Vlaeyen, J. W. S. Does fear of pain moderate the effects of sensory focusing and distraction on cold pressor pain in pain-free individuals? *Journal of Pain* **5**, 250–256 (2004).

37. Schlereth, T., Baumgärtner, U., Magerl, W., Stoeter, P. & Treede, R. D. Left-hemisphere dominance in early nociceptive processing in the human parasylvian cortex. *NeuroImage* **20**, 441–454 (2003).

38. Schrooten, M. G. S., Karsdorp, P. A. & Vlaeyen, J. W. S. Pain catastrophizing moderates the effects of pain-contingent task interruptions. *European Journal of Pain* **17**, 1082–1092 (2013).

39. Seminowicz, D. A., Mikulis, D. J. & Davis, K. D. Cognitive modulation of pain-related brain responses depends on behavioral strategy. *Pain* **112**, 48–58 (2004).

40. Seminowicz, D. A. & Davis, K. D. Interactions of pain intensity and cognitive load: The brain stays on task. *Cerebral Cortex* **17**, 1412–1422 (2007).

41. Slepian, P. M. & France, C. R. The effect of resilience on task persistence and performance during repeated exposure to heat pain. *Journal of Behavioral Medicine* **40**, 894–901 (2017).

42. Stancak, A. *et al.* Neural Mechanisms of Attentional Switching Between Pain and a Visual Illusion Task: A Laser Evoked Potential Study. *Brain Topography* **31**, 430–446 (2018).

43. Terkelsen, A. J., Andersen, O. K., Mølgaard, H., Hansen, J. & Jensen, T. S. Mental stress inhibits pain perception and heart rate variability but not a nociceptive withdrawal reflex. *Acta Physiologica Scandinavica* **180**, 405–414 (2004).

44. Valet, M. *et al.* Distraction modulates connectivity of the cingulo-frontal cortex and the midbrain during pain - An fMRI analysis. *Pain* **109**, 399–408 (2004).

45. Van Damme, S., Crombez, G. & Eccleston, C. Disengagement from pain: The role of catastrophic thinking about pain. *Pain* **107**, 70–76 (2004).

46. Van Damme, S., Crombez, G., Van Nieuwenborgh-De Wever, K. & Goubert, L. Is distraction less effective when pain is threatening? An experimental investigation with the cold pressor task. *European Journal of Pain* **12**, 60–67 (2008).

47. Van Ryckeghem, D. M. L., Crombez, G., Eccleston, C., Liefooghe, B. & Van Damme, S. The interruptive effect of pain in a multitask environment: An experimental investigation. *Journal of Pain* **13**, 131–138 (2012).

48. Van Ryckeghem, D. M. L., Crombez, G., Van Hulle, L. & Van Damme, S. Attentional bias towards pain-related information diminishes the efficacy of distraction. *Pain* **153**, 2345–2351 (2012).

49. Ryckeghem, D. M. L. Van, Damme, S. Van & Vervoort, T. Does attention bias modification training impact on task performance in the context of pain : An experimental study in healthy participants. *PloS one* **13**, e0200629 (2018).

50. Vancleef, L. M. G. & Peters, M. L. The interruptive effect of pain on attention. *Journal of Pain* **7**, 21–22 (2006).

51. Veldhuijzen, D. S., Kenemans, J. L., De Bruin, C. M., Olivier, B. & Volkerts, E. R. Pain and attention: Attentional disruption or distraction? *Journal of Pain* **7**, 11–20 (2006).

52. Verhoeven, K. *et al.* Distraction from pain and executive functioning: An experimental investigation of the role of inhibition, task switching and working memory. *European Journal of Pain* **15**, 866–873 (2011).

53. Verhoeven, K., Goubert, L., Jaaniste, T., Van Ryckeghem, D. & Crombez, G. Pain catastrophizing influences the use and the effectiveness of distraction in schoolchildren. *European Journal of Pain* **16**, 256–276 (2012).

54. Id, Q. C. V., Owen, A., Akin-akinyosoye, K. & Araujo-soares, V. An incremental dual-task paradigm to investigate pain attenuation by task difficulty, affective content and threat value. *PloS one* **13**, e0207023 (2018).

55. Wiech, K. *et al.* Modulation of pain processing in hyperalgesia by cognitive demand. *NeuroImage* **27**, 59–69 (2005).

56. Yamasaki, H., Kakigi, R., Watanabe, S. & Hoshiyama, M. Effects of distraction on pain-related somatosensory evoked magnetic fields and potentials following painful electrical stimulation. *Cognitive Brain Research* **9**, 165–175 (2000).

**Supplementary Methods**

To express in practical terms the trial-by-trial effects of reported thermal sensation and performance on each other, two multilevel general linear models were tested on raw scores for 2-back x Pain condition trials alone. Individual regression slopes and intercepts then were used to estimate within-subject trial-by-trial effects of fluctuations in performance and sensation. First, in order to predict pain differences across an individual's range of 2-back performance, reported sensation was regressed on task performance for each participant. Within each subject, we predicted the pain difference associated with extremes in performance (2-back mean minimal performance A = .59, SD = .20; mean maximal performance A = .98, SD = .03). The mean predicted pain decrement associated with minimal-to-maximal performance is 11.46 points (SD = 37.48). Next, in order to predict 2-back performance differences across participants' ranges of pain report, performance was regressed on reported sensation. Individual mean minimal (67.55 warmth, SD = 40.08) and maximal (153.80, SD = 24.86) reported pain scores were used to predict task performance. The mean predicted task performance decrement associated with minimal-to-maximal pain intensity is (0.04/1.00, SD = 0.16).

In order to examine trial-by-trial interactions between task performance and pain, we performed multilevel mediation analyses on all behavioural trials (MacKinnon, 2008). It has been argued (Zhao, Lynch Jr., & Chen, 2010) that a statistically significant direct effect of the predictor on outcome variable is not required for mediation to apply; therefore, we performed mediation tests regardless of the significance of effects found on subjects-level analyses.

Multilevel analyses resolves violation of the assumption of independence of observations for mediation (MacKinnon, 2008) when examining nested data. Multilevel mediation analyses derive average group-level means (in our case, participant means) and use them to estimate regression slopes and intercepts for nested individual trials. Including second-level moderators allows testing of their contribution to slopes and intercepts within each individual. We expected these analyses to yield results that were slightly different from means-difference analyses, because in the case of multilevel analyses, between-subjects variance is relegated to the 2nd-level rather than conflated with error variance.

Applied to our paradigm, equations for multilevel moderated mediation are:

Y (outcome) predicted by X (predictor)

Trial-level 1:

Y_ij_ = c_j_X_ij_ + e_ij_ (3)

Subjects-level 2:

c_j_ = c_2_Mod_j_ + γ_00_ + u_0j_ (4)

Y predicted by X and M (mediator)

Trial-level 1:

Y_ij_ = c_j_'X_ij_ + b_j_M_ij_ + e_ij_ (5)

Subjects-level 2:

c_j_' = c'_2_Mod_0j_ + γ_00_ + u_0j_ (6)

b_j_ = b_2_ Mod_0j_ + γ_00_ + u_0j_ (7)

M predicted by X

Trial-level 1:

M_ij_ = a_j_X_ij_ + e_ij_ (8)

Subjects-level 2:

a_j_ = a_2_Mod_0j_ + γ_00_ + u_0j_ (9)

Where e is trial-level error variance for the *i* th trial of the *j* th participant, u is subjects-level error variance, γ is grand mean, Mod is the tested subject-level moderator, and a_j ,_b_j ,_c_j ,_c_j_' are trial-level standardized mediation regression coefficients. The values of e, u, β and γ vary across the three main mediation equations, even though notation does not make this explicit. a_2_, b_2_, c_2_ are second-level regression coefficients.

**Example of moderated mediation paths**


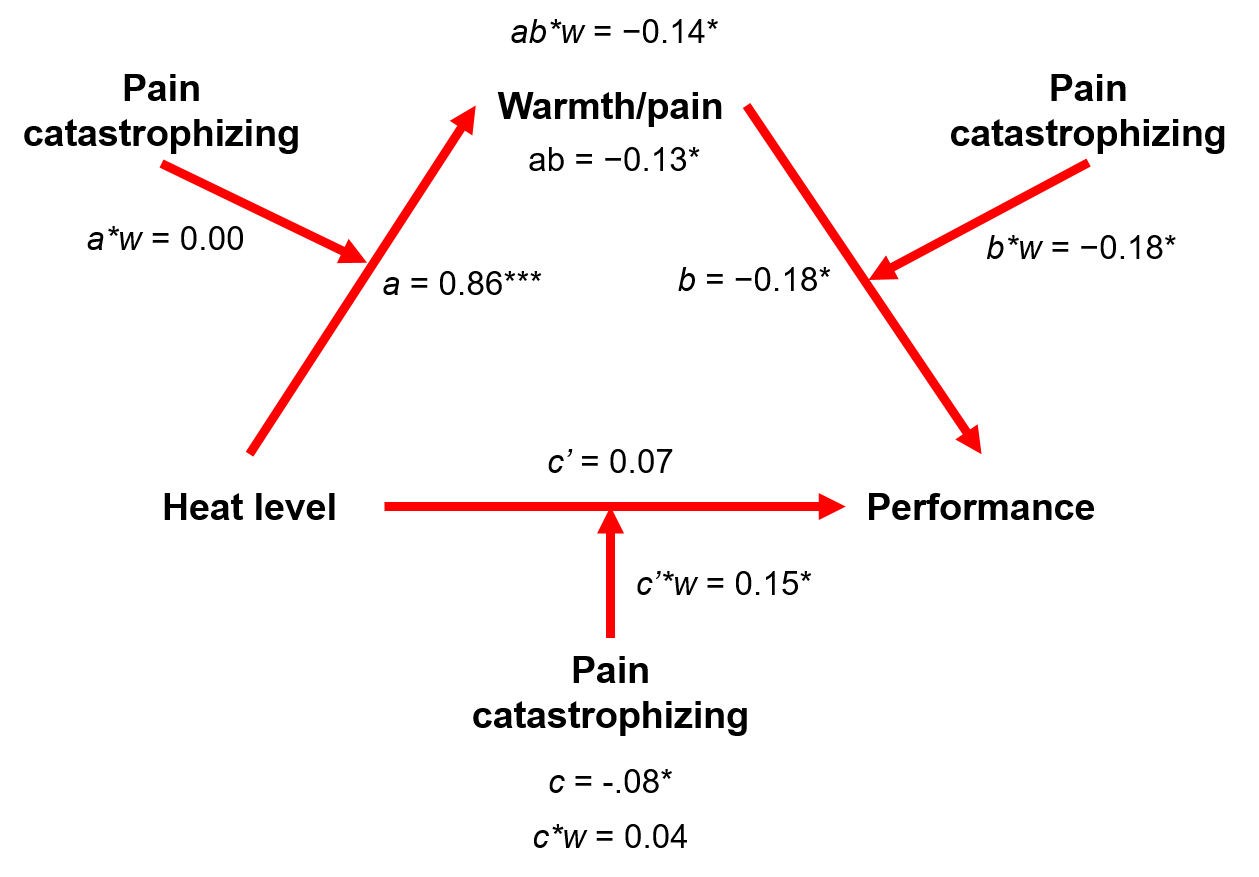


*Supplementary Figure S1.* This figure provides an example of a moderated mediation model using pain catastrophizing scores as a moderator for the paths in the pain interference model (i.e., paths *a*, *b*, *c*, *c′*, and *ab*). A separate model was estimated for each psychological variable of interest (pain catastrophizing, trait anxiety, and trait mindfulness) as the moderator for the pain interference (“red” model) and task analgesia (“blue”) model, respectively. The estimates of the moderated paths for each model can be found in Table 2 of the main text.

**Methods for Figure 4**

In order to graphically illustrate the b-path in the Pain interference model in Figure 2, for each moderator, raw task performance was regressed on raw pain scores in all 2-back trials in a multilevel general linear model analysis, statistically controlling for the effect of heat for both variables. Trial-level coefficients for the effect of pain on performance, as well as coefficients for the effect of subjects-level variables on trial-level coefficients, were derived. For each moderator of interest, the top and bottom quartiles were identified (*n*=10). Given that this analysis z-transforms raw scores before outputting coefficients, in order to obtain x and y intercepts that could be used to plot curves, mean raw pain and performance scores for all 2-back trials were obtained separately for the participants in the top and bottom quartiles for that moderator of interest. For each of the two quartiles plotted, the mean value of the moderator (subjects-level, effect of moderator on first-level parameters) was used to calculate the coefficients of the curve to plot (trial-level, effect of pain on performance), and the x and y intercepts were added to the curve. The line in red depicts the effect of pain on performance for the upper quartile of the moderator, while the black line depicts the relationship for the lower quartile.

In order to graphically illustrate the b-path in the Task Analgesia model, for each moderator, raw pain scores were regressed on raw performance in all high heat trials, statistically controlling for the effect of task difficulty for both variables. Trial-level coefficients for the effect of performance on pain, as well as coefficients for the effect of subjects-level variables on trial-level slopes and intercepts, were derived. For each moderator of interest, the top and bottom quartiles were identified (*n*= 10). In order to obtain x and y intercepts for plotting lines, mean raw pain and performance scores for all 2-back trials were derived separately for the participants in the top and bottom quartiles for that moderator of interest. For each of the two quartiles plotted, the mean value of the moderator was used to calculate the coefficients of the line to plot (effect of performance on pain), and the x and y intercepts were added to the curve. The line in blue depicts the effect of performance on pain for the upper quartile of the moderator, while the black line depicts the relationship for the lower quartile.
